# Universal dynamic fitting of magnetic resonance spectroscopy

**DOI:** 10.1002/mrm.30001

**Published:** 2024-01-24

**Authors:** William T. Clarke, Clémence Ligneul, Michiel Cottaar, I. Betina Ip, Saad Jbabdi

**Affiliations:** Wellcome Centre for Integrative Neuroimaging, FMRIB, Nuffield Department of Clinical Neurosciences, University of Oxford, Oxford, UK

**Keywords:** dMRS, edited-MRS, fMRS, MRS, spectroscopy

## Abstract

**Purpose:**

Dynamic (2D) MRS is a collection of techniques where acquisitions of spectra are repeated under varying experimental or physiological conditions. Dynamic MRS comprises a rich set of contrasts, including diffusion-weighted, relaxation-weighted, functional, edited, or hyperpolarized spectroscopy, leading to quantitative insights into multiple physiological or microstructural processes. Conventional approaches to dynamic MRS analysis ignore the shared information between spectra, and instead proceed by independently fitting noisy individual spectra before modeling temporal changes in the parameters. Here, we propose a universal dynamic MRS toolbox which allows simultaneous fitting of dynamic spectra of arbitrary type.

**Methods:**

A simple user-interface allows information to be shared and precisely modeled across spectra to make inferences on both spectral and dynamic processes. We demonstrate and thoroughly evaluate our approach in three types of dynamic MRS techniques. Simulations of functional and edited MRS are used to demonstrate the advantages of dynamic fitting.

**Results:**

Analysis of synthetic functional ^1^H-MRS data shows a marked decrease in parameter uncertainty as predicted by prior work. Analysis with our tool replicates the results of two previously published studies using the original in vivo functional and diffusion-weighted data. Finally, joint spectral fitting with diffusion orientation models is demonstrated in synthetic data.

**Conclusion:**

A toolbox for generalized and universal fitting of dynamic, interrelated MR spectra has been released and validated. The toolbox is shared as a fully open-source software with comprehensive documentation, example data, and tutorials.

## 1 Introduction

During dynamic, or 2D, MRS, multiple spectra are acquired while experimental conditions change. Dynamic changes can be induced deliberately, to sensitize acquisitions to different signal mechanisms. Conditions can also change due to uncontrollable physiological processes, such as structured noise from cardiorespiratory motion or voluntary movement,^1^ or due to hardware drift.^2^ In all types of dynamic MRS, the classical processing pipelines start by fitting a spectral model to each transient,^3,4^ or to averages of repeated measurements.^5,6^ They then extract parameters of interest from these fits, usually metabolite concentrations, and analyze or model their changes across experimental conditions. However, MRS is an inherently low SNR technique compared to proton-MRI, as metabolites occur with concentrations thousands of times lower than water. This means repeated measurements are required, at the detriment of more interesting and informative changes induced experimentally. Simultaneous fitting of all spectra, i.e., dynamic fitting, can mitigate this trade-off, by modeling the effect of changing the experimental conditions and by explicitly sharing relevant information across dynamic spectra.

For example, in spectral editing,^7^ two or more spectra are acquired with appropriate changes in the pulse sequence aimed at suppressing the signal around targeted spectral peaks. While these spectra may be affected by different factors that require separate modeling, such as phase shifts,^8^ they share the fact that the underlying metabolite concentrations are unaffected by the pulse sequence. A straightforward dynamic fit can estimate the shared concentrations while independently fitting nuisance factors. A similar logic applies for non-edited spectroscopy, where noisy transients are affected by separate artifacts while metabolite concentrations remain constant.^2^ In diffusion-weighted MRS,^6^ the apparent concentrations are reduced as a function of the diffusion encoding due to the random motion of metabolites.^9^ Models linking metabolite diffusion to the underlying tissue microstructure can be used to link across dynamic spectra,^10^ thereby imposing a precise structure to help the fitting, particularly when strong diffusion-encoding drastically decreases signal-to-noise. A similar approach can be used in functional MRS,^5,11^ where the experimental manipulation is usually an exogenous stimulus, which effect on the concentrations and potentially on other parameters such as the linewidth can be explicitly modeled.^12^

The possible applications of dynamic fitting are almost universal. They are not limited to the aforementioned example areas, but include all but the most basic acquisition paradigms, for example: metabolic kinetic measurements in phosphorus MRS,^13^ deuterium metabolic imaging,^14^ and time-resolved carbon imaging (both hyperpolarised^15^ and non-hyperpolarised^16^); “Fingerprinting” techniques which aim to elucidate multiple metabolic state parameters^17^; and multi-inversion and multi-echo techniques that measure relaxation parameters,^18^ which might be sensitive markers of pathology.^19,20^ There may also be ample application for the better processing of even straightforward acquisition paradigms.^1^

**Figure 1:**
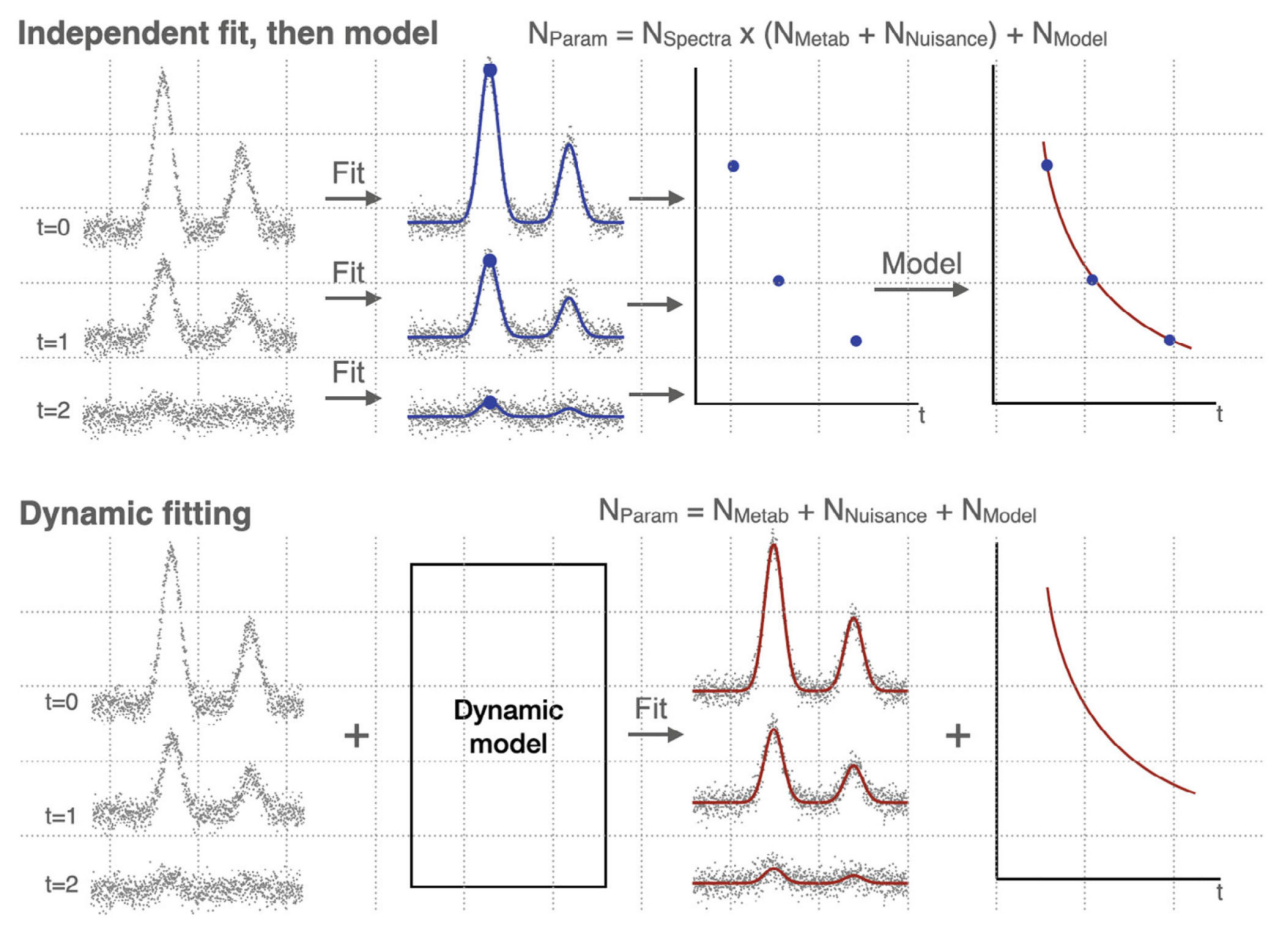
Typical current independent fitting of dynamic data vs. proposed dynamic fitting. The typically used approach in fitting a model to dynamic MRS data (top) is to model the changing parameters after an independent spectral fitting stage (where each spectrum is treated independently). The proposed approach (and as examined by Tal^34^) is to simultaneously fit a spectral and dynamic model. This is known as dynamic, “2D,” or *spectral-temporal fitting*. This approach reduces the number of parameters to fit by allowing estimation of shared model parameters at once. This shared estimation increases the amount of data used to estimate parameters that are expected to be static (or functionally linked) across transients, mitigating the effect of noise which would otherwise result in multiple, low precision estimates of the parameter. This results in a decrease in parameter uncertainty. N_Param_: Total number of fitted parameters, N_Metab_: number of metabolite concentration parameters, N_Nuisance_: number of spectral fitting parameters not of direct interest (e.g., line broadening), N_Model_: number of dynamic model parameters.

Dynamic fitting has repeatedly been demonstrated as advantageous over independent “1D” fitting for specific use cases: initially for T_1_ determination by inversion recovery,^21^ and then incorporating T_2_ measurements as well,^21,22^ and even with extensions for determining unknown macromolecule components^18,23,24^; in fitting 2D j-resolved spectroscopy^25,26^; in fitting time resolved x-nuclear data^27^; fitting edited data^28^; and diffusion,^29^ including more complex, non-Gaussian diffusion models^30^; and even extends to multi-voxel MRSI.^31^ In some of these cases, software has been released for these specific uses.^24,25,29^ However, in this work we introduce an extension to FMRIB software library (FSL)-MRS^32^ that allows direct fitting of an arbitrary dynamic signal model to multiple spectra simultaneously. FSL-MRS is an end-to-end spectroscopy analysis toolbox embedded in FSL (a neuroimaging package).^33^ Using a dynamic model for spectral fitting of multiple signal transients reduces the number of parameters to be estimated from noisy data (Figure 1), and, as demonstrated by the previously listed works and, in a general case, by Tal,^34^ reduces fitting uncertainty. (The latter demonstration is replicated in this work.) These enhancements also establish a framework for model selection, as well as robust statistical testing at the group-level. Although similar tools have been implemented, they have focused on specific and limited use cases^24,25,29^; the toolbox described here is open source, freely available, and importantly, allows arbitrary model flexibility for arbitrary types of dynamic MRS experiments.

Here, we demonstrate uses across in vivo and simulated data of three contrasts to evidence the suitability of our free, open toolset. These contrasts are:

1 Spectral editing of the metabolite gamma-aminobutyric acid (GABA) (MEGA-PRESS^8^),
2 Functional MRS (fMRS) measured during visual stimulation, and,
3 Diffusion-weighted MRS (dMRS).

We show the value of dynamic fitting in improving parameter estimation and uncertainty in a general framework, before extending the analysis to simulation of real-world fMRS data. With spectral editing, we show improvements in precision (the underlying variance of the estimated parameters), with fMRS, we validate the accuracy (the bias or difference to a ground truth) of the implementation and demonstrate how the toolset can be used to mitigate confounds and unlock new measurement approaches. As a further validation, we replicate the results of two in vivo studies (fMRS and dMRS respectively) using their original data.

## 2 Methods

### 2.1 Approach

In this work, we use six case studies (CS) to explore analysis of common dynamic MRS contrasts. Each case study explores a particular contrast, while either validating the dynamic approach and toolset, or highlighting an advantage over current processing approaches. Each case study is presented with its setting (i.e. methods) and outcome (i.e. results) in the same subsection of the Results section. The six case studies are:

1 **fMRS: replication and extension of Tal’s monograph**—shows improved accuracy and precision of correlated parameters in fMRS,
2 **Edited-MRS: improved estimation of [GABA]**—further reveals improved fitting error in metabolite concentrations when dynamic fitting is used,
3 **fMRS: simulated analysis and group statistics**—demonstrates how an analysis of visual stimulation fMRS can be performed, through to group-level statistics,
4 **fMRS: in vivo confound mitigation**—explores the ability to dynamically model blood-oxygen-level-dependent signal as a confound in the fMRS model in real data,
5 **dMRS: multi-direction diffusion encoding**—shows how dynamic fitting allows higher dynamic encoding resolution than would otherwise be restricted by SNR,
6 **dMRS: in vivo validation**—demonstrates a full study analysis using analytical diffusion signal representations, with different models applied to different metabolites.

Our approach is to show that dynamic fitting reduces error when model parameters are correlated, as predicted by Tal (and shown in CS1 and CS2). We also show that FSL-MRS dynamic fitting advances the analysis approach by either: providing a robust statistical framework (CS3), mitigating confounds (CS4 and CS6), or extending the available acquisition approaches (CS5). CS3 can also be used as a fully featured toolset demonstration, and the results as an implementation validation of the tool.

### 2.2 Model

We describe the evolution of model parameters as a “time dependence,” irrespective of how experimental conditions change. We use the linear combination spectral fitting model of FSL-MRS,^32,35^ modified to allow time dependence for all model parameters:

(1) $$\begin{array}{l} s(\nu ,t)=B(\nu ,t)+e^{-j\left({\text{Φ}}_{0}(t)+\nu {\text{Φ}}_{1}(t)\right)}\\ \;\times \underset{k}{\sum }c_{k}(t)ℱ\left[m_{k}(\tau ,t)e^{-\tau (\gamma (t)+j\epsilon (t))}\right]. \end{array}$$

The time dependence of the concentration *c*_*k*_, line-shape *γ*, shift *ϵ*, phase **Φ**, and baseline *B* parameters are specified in an editable, Python language, configuration file. Each of the parameters can have their own arbitrary dynamic models to describe their time-dependent behavior. Parameters may be fixed across all time-points, estimated per time point, or constrained to an analytical or numerical model across time. When combined with the ability to specify a linear combination basis set of spectra per time-point, the configuration file approach enables the description of many different types of dynamic MRS. Each dynamic model can come with its own set of dynamic parameters, which are estimated alongside the spectral parameters using all the data at once. More details on model initialization and fitting can be found in the Supporting Information S1.

### 2.3 Dynamic model specification

The joint spectral-dynamic model is specified through a user-defined configuration file which details the choices of dynamic fitting and the associated dynamic parameters. The user can also specify a time variable input, which contains information about the experimental design leading to dynamic changes. For example, the b-values and gradient directions for dMRS, or a stimulus design matrix for fMRS. The core spectral fitting model is then specified in the same way as a normal linear combination model, as in non-dynamic FSL-MRS fitting.

The Configuration File is simply a Python language text file containing:

1 The time-dependent behavior of each spectral parameter: fixed, fully variable, or model-constrained,
2 Fitting bounds for each free parameter (optional),
3 Arbitrary dynamic-model definitions as python-language functions.

Time-dependence may be defined for a sub-group of parameters, e.g., metabolite concentrations or FSL-MRS “metabolite-groups” (which link frequency shift and line broadening parameters). Dynamic model functions must also provide analytical or numerical gradient definitions in the configuration file. Example configuration files are included in Figure S1.

### 2.4 Higher-level/group analysis

FSL-MRS implements python scripting (fsl_mrs.utils.fmrs_tools) and command line (fmrs_stats) interfaces to carry out higher-level or group-level analysis. These tools constitute a Python wrapper around the FSL tool FLAMEO, which implements multilevel linear modeling for group analysis using Bayesian inference.^36^ The tools allow the formation of both first level linear contrasts and high level (group) contrasts, and includes the ability to combine metabolites when the underlying first-level dynamic model is linear.

### 2.5 Software

Our universal dynamic fitting toolbox is implemented as part of the FSL-MRS spectroscopic analysis package (part of the FMRIB Software Library, FSL^33^), available free of charge for academic use, and published as open-source. Dynamic fitting may be run in Python coding environments (using the sub-package *fsl_mrs.dynamic*) or by using the command-line scripts *fsl_mrs_dynamic*, and *fmrs_stats*. Documentation is provided in the source-code repository and at fsl-mrs.com.

FSL-MRS is open-source, with code available online at git.fmrib.ox.ac.uk/fsl/fsl_mrs. Version 2.1.0 of FSL-MRS was used, permanently available at Reference 37. All code and data used in generating this manuscript are available online at git.fmrib.ox.ac.uk/wclarke/fsl-mrs-dynamic-fitting (#11e4d66f), and permanently available online at Zenodo.^38,39^

### 2.6 Software outputs

Results of the fitting are output as data tables comprising fitted free parameters, estimated uncertainty on the free parameters, the covariances between each free parameter, their interpretation as mapped parameters, and the results of the initialization. Fitting quality parameters, such as the Akaike Information Criterion are also output. Visualization of the results and fit quality is provided either via the Python API, or as an interactive HTML results summary document. Examples of the visualizations are provided in the Supporting Information S1 (Example Data Visualization section).

## 3 Results

### 3.1 CS1. Functional MRS: replication, and extension of Tal

Recently the advantages of dynamic fitting of 2D data (also called spectral-temporal fitting) were demonstrated theoretically and numerically.^34^ Here, we replicated these results using the software framework of FSL-MRS and extended the simulations from toy (two resonance) examples to realistic ^1^H-fMRS data, containing many overlapping spectral resonances. Functional MRS temporally resolves MRS to detect changes in neurochemical concentrations (or metabolite visibility), induced by external sensory stimulus or otherwise evoked neural activity.^11^

#### 3.1.1 Setting

The first simulation implements Tal’s^34^ toy example. It uses 64 repetitions of a spectrum containing two Lorentzian peaks at defined, but variable separation (Figure S3). For the central half (32 repetitions) one peak increased in amplitude by 20%, the other peak remained constant throughout (Figure S2). This toy simulation of fMRS was fitted using the FSL-MRS dynamic approach implementing a general linear model (GLM) to model the temporal dynamics. A design matrix with two regressors (baseline and rectangular-function stimulation period) was used. For comparison, the data were also fitted using FSL-MRS’s independent spectral fitting routine, and the GLM then fitted to the concentration parameters extracted from the independent spectral fits (as in Figure 1). Each simulation was generated and fit 100 times for each of 10 peak separations and three different SNR levels. Fitting was carried out as in the original publication of *Tal*, with two peaks that are fitted with independent concentrations, frequency shifts, and linewidths, “Free,” and then using the normal FSL-MRS fitting model, with fixed frequency offsets (i.e. one global shift for both peaks) and a single linewidth (both peaks broadened with one linewidth parameter), “Linked.”

**Figure 2:**
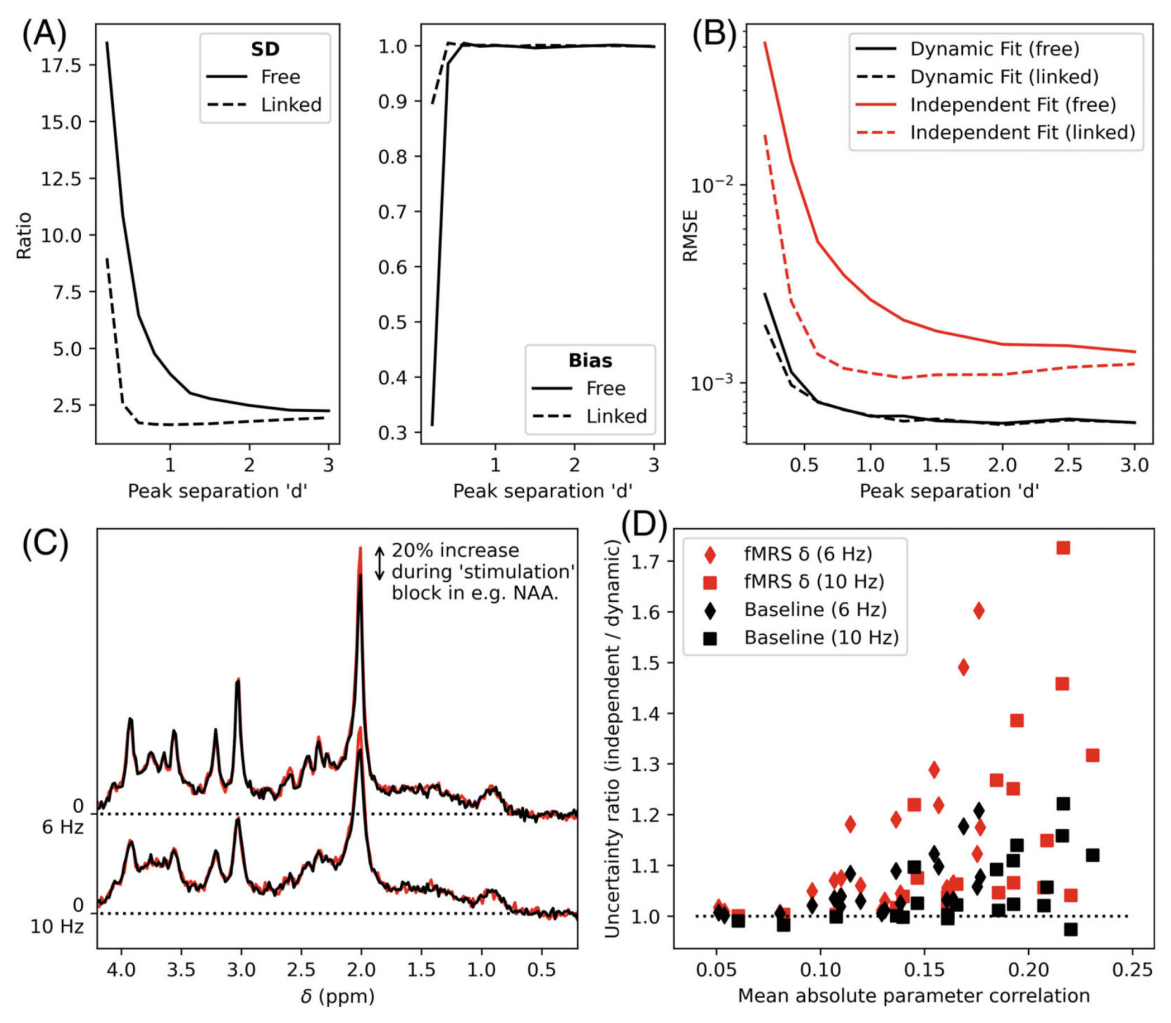
Results of the functional MRS validation. (A) Ratio of Monte Carlo measured SDs and bias (independent fitting/dynamic fitting) for the concentration increase as a function of peak separation in the toy two-peak simulation (see Figure S3). Results for a model with all parameters unlinked “Free” and the standard FSL-MRS fitting model “Linked” are given (see §Functional MRS–Simulation). (B) RMSEs for the same simulation. As shown in A&B Dynamic fitting reduces uncertainty and overall error. (C) Extension of fMRS validation to realistic ^1^H-MRS data. Paired data with 20% increases in concentration were simulated for each metabolite (NAA shown) at two linewidths. (D) The uncertainty ratio (ratio of SDs, independent fitting/dynamic fitting) for each metabolite’s baseline concentration and increase (delta) is shown as a function of the parameter’s mean correlation with other parameters (see Figure S4). A value >1 indicates that dynamic fitting is decreasing the uncertainty compared to independent fitting.

For each separation, the estimated amplitude increase was extracted (specified in the GLM as the beta for the rectangular-function stimulation regressor). The RMS error (RMSE) across all repetitions was calculated for the independent and dynamic fits, and the ratio of the uncertainties was calculated (ratio of SDs, for both free and linked conditions, Figure 2A,B).

This section’s code is contained in the online repository under. /fmrs/1_two_peak_simulation (git.fmrib.ox.ac.uk/wclarke/fsl-mrs-dynamic-fitting/-/tree/master/fmrs/1_two_peak_simulation).

The second simulation extended the above approach to realistic spectral profiles. In addition, noting that peak separation is a key driver of parameter correlation, the simulation was carried out at two different linewidths (6 and 10 Hz). As such, the same overall approach was taken as the first simulation but implemented with simulated 3T proton magnetic resonance spectroscopy (^1^H-MRS) spectrum from the brain. For each of 20 metabolites in the spectrum (see Supporting Information S1), and for each of the two linewidths, 500 Monte Carlo repetitions were made where one specified metabolite in each simulation increased in amplitude by 20% (example for NAA in Figure 2C) for the central 30 repetitions of 60 total repetitions. All other metabolites were held constant for that case, with the specified metabolite changed for each subsequent case.

Fitting was carried out as for the previous simulation with dynamic fitting implementing a GLM dynamic model using a design matrix with two regressors. No BOLD-like effects on linewidths were simulated.^12^

For each metabolite (and linewidth case) the ratio of independent/dynamic fitting uncertainties (calculated as the SD cross the 500 Monte Carlo repetitions) was calculated for the baseline and stimulation regressor beta. The mean correlation between all other fitting parameters and each concentration parameter was calculated as described in Figure S4.

This section’s code is contained in the online repository under . /fmrs/2_fmrs_spectrum_simulation (git.fmrib.ox.ac.uk/wclarke/fsl-mrs-dynamic-fitting/-/tree/master/fmrs/2_fmrs_spectrum_simulation).

#### 3.1.2 Outcome

The first simulation (toy two-peak) shows that in all cases the dynamic fitting approach reduces the uncertainty of the amplitude increase parameters, bias was equal for all separations except the smallest separation, where independent fitting was more accurate on average. Combining the two using RMSE, dynamic fitting always outperformed independent. The functional form of the uncertainty ratio as a function of peak separation “d” replicates that found by Tal (figure 4 in Tal).^34^ Linking the linewidth and shift parameters, as is done in the default FSL-MRS model, reduces the advantage of dynamic fitting.

The second simulation (realistic ^1^H-MRS spectrum) also demonstrates the advantage of using dynamic fitting over independent fitting for estimating both fMRS amplitude changes and also underlying baseline concentrations. A clear relationship between mean parameter correlation and uncertainty reduction was observed, with wider linewidths giving higher correlations and larger improvements (Figure 2D). This is also observed in the differences between the “Linked” and “Free” implementations of the first simulation: the effect of linking the peak’s linewidths and shifts removes a major source of parameter correlation, reducing the advantages of dynamic fitting.

### 3.2 CS2. Edited-MRS: improved estimation of [GABA]

#### 3.2.1 Setting

**Figure 3:**
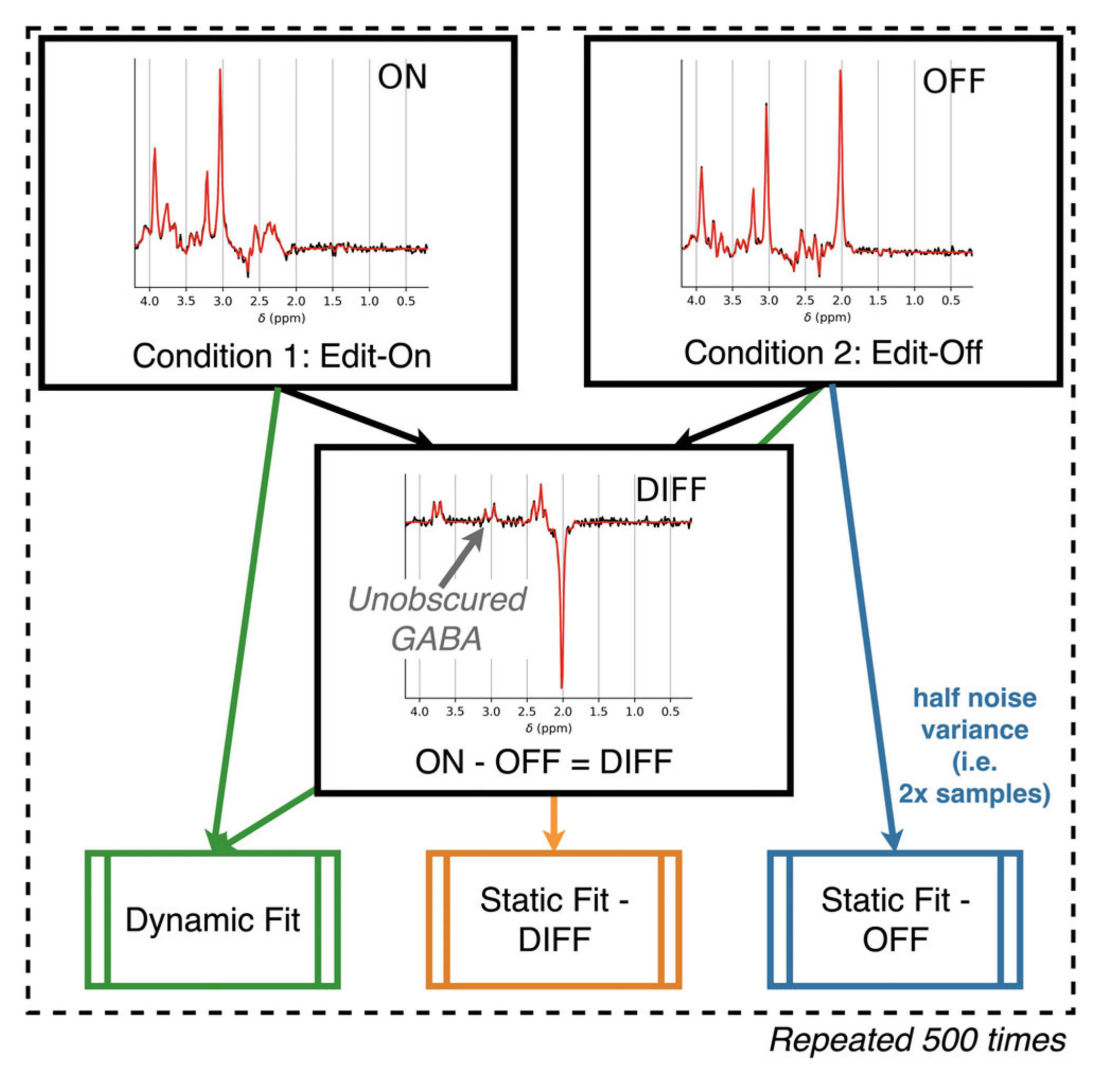
Approach to edited MRS analysis simulation. Simulation is carried out by generating pairs of synthetic MEGA-edited spectra (both the edit-off [OFF] and edit-on [ON] saturation case), and the corresponding difference spectrum (by subtraction, [DIFF]). The DIFF and OFF spectra are fit using single spectrum fitting, and the ON + OFF spectra are fit using the dynamic approach. The statically fitted OFF spectrum is constructed with half the noise variance to simulate matched time acquisitions. This is repeated 500 times in a Monte Carlo approach for each noise level and line broadening. Spectra are shown with static fitting and have the lowest linewidth (5 Hz) and intermediate noise (noise SD = 144).

The second case study uses synthetic single voxel MEGA-PRESS data.^8^ This example is representative of a study that acquires data using the MEGA-PRESS sequence, in absence of an external stimulation paradigm, to measure the concentration of metabolites (e.g. GABA) that are obscured by, or highly correlated with, other metabolite signals. MEGA-PRESS acquires two encoding conditions (ON and OFF), the difference of which (DIFF) contains a simplified spectrum enabling unobscured estimation of GABA (Figure 3). Here, the accuracy and precision on measurements of metabolites (specifically: NAA, creatine, GABA and Glx [glutamate plus glutamine]) are compared across three fitting strategies:

1 “OFF”—Edit-off-only acquisition—Using only the edit-off saturation condition, without a subtraction stage. All metabolites are visible but many overlap. The spectrum is fitted with a single (unmodified) set of basis spectra.
2 “DIFF”—Forming a difference spectrum—This approach matches the current gold-standard approach. An edit-off saturation condition is subtracted from an edit-on saturation condition to leave a spectrum containing the differences arising from j-coupling (and direct saturation effects). The difference spectrum is fitted using a modified set of basis spectra.
3 “DYN”—Dynamic fitting of edit-off and edit-on acquisitions—The proposed approach, edit-off and edit-on saturation conditions are used in analysis, but no subtraction is performed, and they are analyzed together using the proposed simultaneous fitting approach. In this case each spectrum is fit with a relevant set of basis spectra (simulated with edit-off and edit-on saturation) with additional dynamic constraints. These constraints are equal metabolite concentrations and nuisance parameters (lineshape, shift, phase, baseline, etc.).

In each case the total acquisition time was kept constant, that is, case one (edit-off-only) data were simulated with half the noise variance. Basis sets were simulated using FSL-MRS’s simulator (fsl_mrs_sim). The difference basis set was constructed from the subtraction of the OFF from the ON basis set, which respectively simulated editing pulses at 7.5 and 1.9 ppm.

Data were simulated for standard in vivo concentrations for 19 metabolites (specified, with concentrations, in the Supporting Information S1), no macromolecules were simulated. Data were simulated with Lorentzian linewidths (FWHM) in four steps from 5 to 9 Hz (representing “excellent” to “acceptable” linewidths as defined in Juchem et al.),^40^ and eight SNR levels (NAA singlet SNR, measured with an exponential matched filter^41^; SNR of 30–330 in eight steps) that span (and extend beyond) the range observed in vivo. Each condition was simulated 500 times to carry out Monte Carlo sampling of the fitting process. Data were fit using FSL-MRS’s core fitting routine fit_FSLModel (parameters specified in Data S1) or the dynamic fitting approach as detailed for fitting case #3. Simulation code for this section is contained in the online repository under. /editing (git.fmrib.ox.ac.uk/wclarke/fsl-mrs-dynamic-fitting/-/tree/master/editing).

#### 3.2.2 Outcome

**Figure 4:**
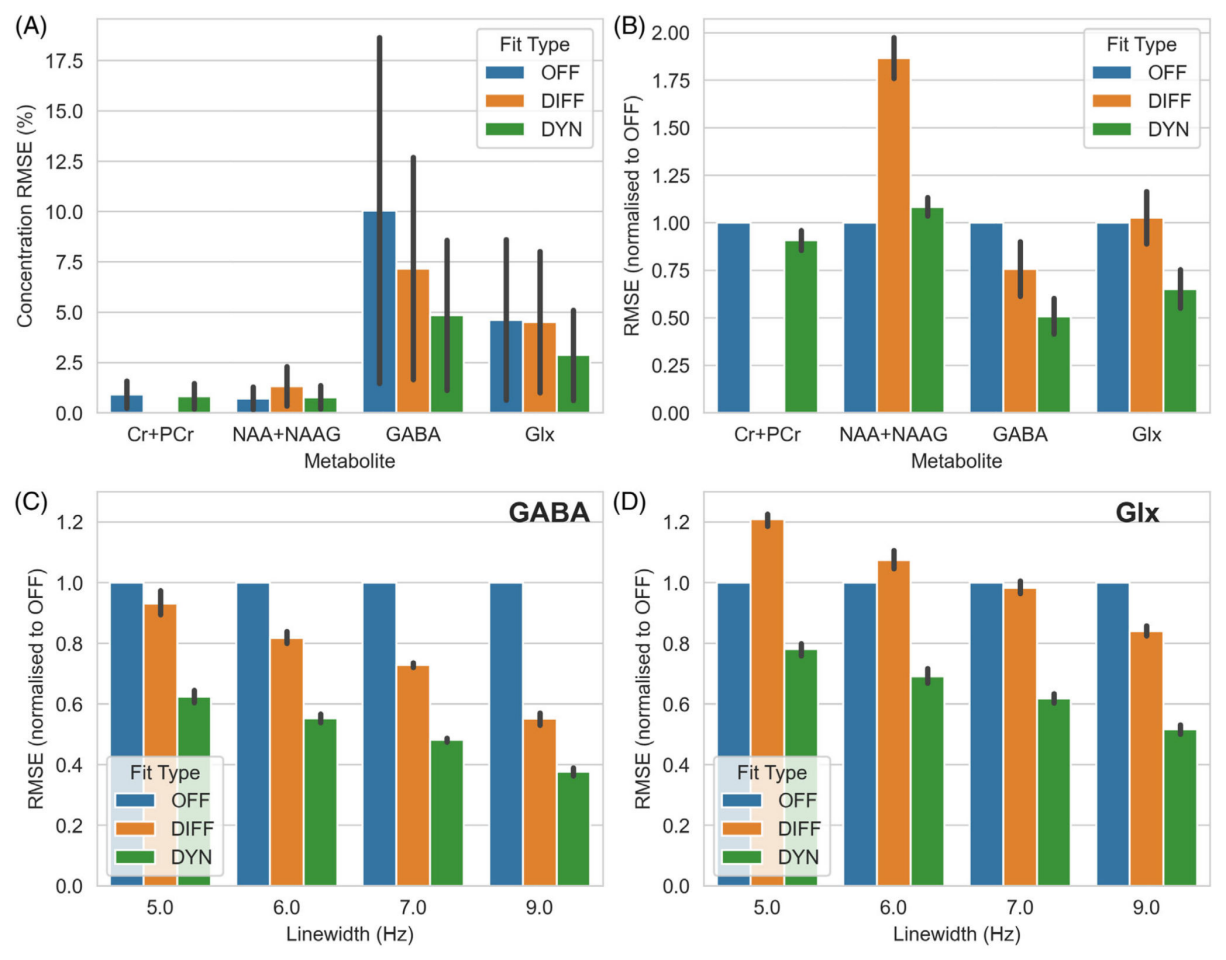
Results of the editing simulation. (A) RMSE (±SD) across all noise levels and linewidths for each examined metabolite, expressed as percentage of the true metabolite concentration. (B) As (A), but with the results normalized to OFF for each metabolite. (C, D) The effect of linewidth on the relative performance for GABA and Glx (glutamate + glutamine). In all cases, except the measurement of NAA + NAAG, the RMSE is lowest for the dynamic approach.

For each metabolite and each fitting condition (#1–3) the RMSE was calculated across all Monte Carlo repetitions. RMSE was expressed in both concentration units (equivalent to mM) or normalized to condition #1 (edit-off-only). Results from four representative metabolites are shown (Figure 4): the usual targets of MEGA editing, GABA and Glx, a metabolite that appears in all conditions, tNAA (NAA + NAAG), and one which is removed in the differencing process, tCr (creatine + phosphocreatine). Results are also shown for four different linewidths for GABA and Glx (Figure 4C,D).

For GABA, RMSE was always worst (highest) for OFF (#1), then DIFF (#2) and the lowest was the proposed method DYN (#3). The greatest improvement for DIFF or DYN was seen for widest (worse) linewidths, with DYN achieving an RMSE of 0.39 of the OFF condition with a linewidth of 9 Hz compared to 0.6 for 5 Hz. Across all linewidths DYN achieved a 33% reduction in GABA RMSE compared to DIFF. A similar relationship was seen for Glx, except DIFF was the worst performing fit strategy with narrow linewidths, DYN was always the best.

For tCr and tNAA, DYN produced highly similar results to OFF, both of which substantially outperformed DIFF. No significant variation was observed as a function of SNR or of linewidth for tCr or tNAA (Figures S5 and S6).

### 3.3 CS3. fMRS: simulated analysis and group statistics

#### 3.3.1 Setting

**Figure 5:**
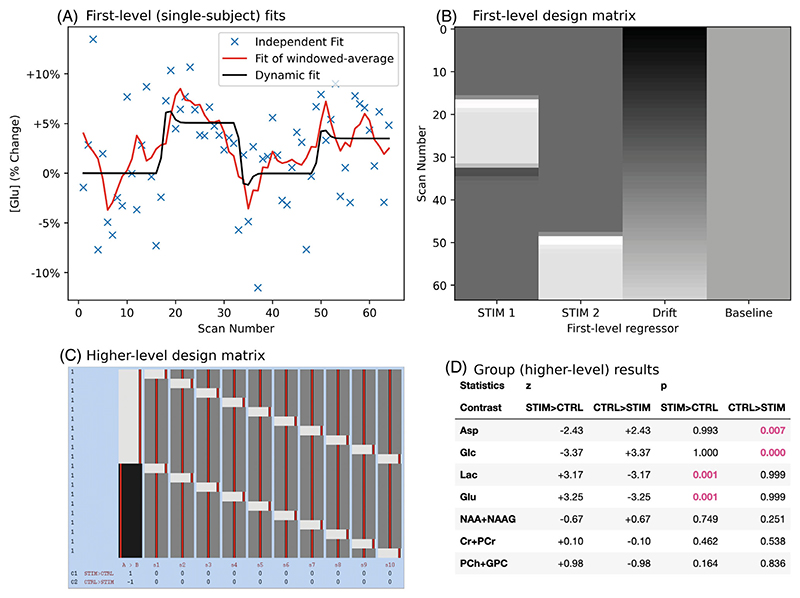
Subject and group analysis of functional MRS using a GLM. (A) Single subject fit of glutamate. A single subject’s stimulation data are shown for relative glutamate changes. Independent, moving-window temporally smoothed, and dynamically modeled results are shown. Note that no formal comparison is made to the moving-window method, comparison of dynamic fitting to independent fitting is made in CS1. (B) Design matrix used to both generate and fit the fMRS data at the first level. There are two stimulation regressors, a linear drift regressor and a constant regressor. (C) The group level analysis used this design matrix to run a paired t-test for stimulation and control (no effect) datasets. Each row indicates one scan (control or stimulation) with all stimulation listed first, columns show each explanatory variable with a value of −1 (black), 0 (dark gray), or 1 (gray). The matrix was created and displayed using FSL tools as explained at Reference 42. (D) Output of FSL-MRS’s fmrs_stats tool showing group z and p statistics for each first-level contrast. The tool accurately identifies the metabolites changing in the simulation as significant.

A full set of simulated visual-stimulation data and analysis scripts has been created for the purpose of demonstrating fMRS analysis using the proposed dynamic fitting approach. The data simulate single-voxel data acquired using block visual stimulation at 7T, in 10 subjects, with a separate stimulation and control condition for each subject. Metabolite concentration changes, inter-subject variance in concentration changes and spectral quality is matched to reported values.^12^ As such, glutamate and lactate were set to increase during stimulation and glucose and aspartate to decrease, on average all other metabolites should be constant. Line narrowing due to the positive BOLD effect was simulated. The input dynamic model uses the canonical BOLD hemodynamic response function to model all changes (metabolite concentrations and line narrowing) during the stimulation period, and is implemented in a design matrix for GLM with four regressors (two stimulation conditions, linear drift, and a constant for modeling baseline concentration, Figure 5B). The simulation implementation is detailed in the Supporting Information S1. Group level analysis was conducted using the fmrs_stats function from FSL-MRS, implementing a paired t-test design across the stimulation and control datasets.

This documented demonstration dataset and analysis is hosted separately at github.com/wtclarke/fsl_mrs_fmrs_demo, with a permanent record at GLM-FslWiki.^43^ In addition to demonstration, this dataset was used to assess the implementation accuracy of the proposed dynamic fitting for fMRS combined with the packaged MRS group-level statistics tool (fmrs_stats). To assess the implementation accuracy, the betas for each concentration related regressor was compared to the true simulation input value, per subject and as a group average. Code relevant to this validation is contained under /fmrs/3_fmrs_demo (git.fmrib.ox.ac.uk/wclarke/fsl-mrs-dynamic-fitting/-/tree/master/fmrs/3_fmrs_demo).

#### 3.3.2 Outcome

**Figure 6:**
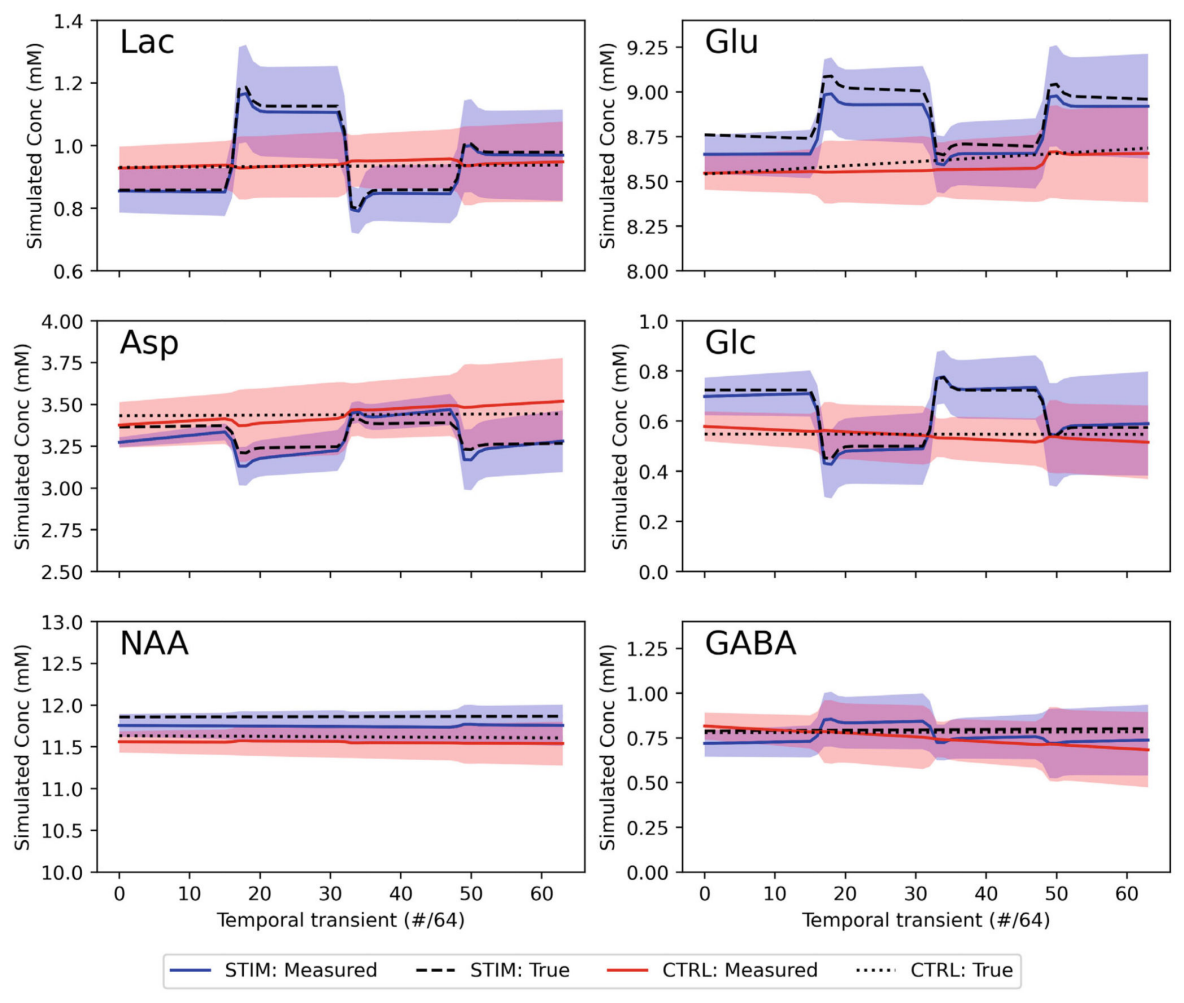
Visual representation of functional mrs (fMRS) demonstration group-level results. Stimulation and control (no stimulation) cases are plotted as a function of temporal transient showing group mean and SD. The true values are shown as dashed/dotted lines. All metabolites that had simulated changes (Lac & Glu—positive, Asp & Glc—negative) are shown alongside two with non-changing metabolites (NAA—high SNR, GABA—low SNR).

Implementation accuracy was assessed by comparing GLM betas related to metabolite concentrations (constant and stimulation terms) per subject and at the group level. Figure 6 shows the graphical outputs of group level traces for major metabolites (with Lac, Glu, Asp, and Glc expected to change).

Correlation and Bland–Altman analysis of individual concentration betas is presented in the Supporting Information (Figures S7 and S8) and summarized here. Measured betas showed a very high level of correlation at both individual subject level (Pearson’s *r* = 0.98, bias = 0.4 mM) and group (Pearson’s *r* = 0.91, bias = 0.02%).

### 3.4 CS4. fMRS: in vivo confound mitigation

#### 3.4.1 Setting

The proposed dynamic fitting approach was assessed by reanalyzing previously published visual stimulation fMRS data.^44^ The original study implements commonly applied analysis approach of carrying out independent spectral fitting on temporally averaged data, and then correlating metabolite time courses (which are themselves further smoothed). The dataset comprises 13 subjects scanned for 8.5 min per condition. Two conditions were acquired: a stimulation condition, “eyes-open,” with four blocks of flashing checkerboard visual stimulation presented for 64 s interleaved with 64 s rest (no stimulation) blocks, and a control condition with “eyes-closed” (no stimulation presented). Signal was acquired using a sLASER sequence interleaved with 3D EPI, full details are available in the original publication^44^ and are summarized in the Supporting Information S1. The interleaved EPI data of the original dataset were not used in this reanalysis. Human data were collected with informed, written consent, approved by the University of Oxford Research Ethics Committee (MSD-IDREC-C1-2014-146).

Spectral pre-processing was carried out using FSL-MRS’s pre-processing routine, fsl_mrs_preproc. The data were analyzed using a dynamic fitting approach implementing a GLM model for dynamic analysis. The design matrix was implemented with four stimulation regressors, a linear drift and constant term, generated using the Glover HRF in the Nilearn package.^45,46^ The spectral fitting component used the original study’s basis spectra set (further parameters are listed in the Supporting Information S1). Two different approaches to BOLD-induced line narrowing were tested, one where the linewidths were kept fixed across time, and one where the Lorentzian line-broadening was modeled as a GLM (using the same design matrix as metabolite concentrations). This aims to remove the step of applying line broadening to spectra during stimulation events, as carried out in the original study (and others).^12,44^ Applying line broadening will result in autocorrelation of spectral points and modify noise properties in just the stimulation case.

Group level analysis was carried out using FSL-MRS’s fmrs_stats routine implementing a paired t-test design across stimulation and control conditions for each subject (as in CS3). Group results were compared with the original study’s findings.

Code for this section is contained in the online repository under. /fmrs/4_fmrs_invivo_example (git.fmrib.ox.ac.uk/wclarke/fsl-mrs-dynamic-fitting/-/tree/master/fmrs/4_fmrs_invivo_example).

#### 3.4.2 Outcome

**Figure 7:**
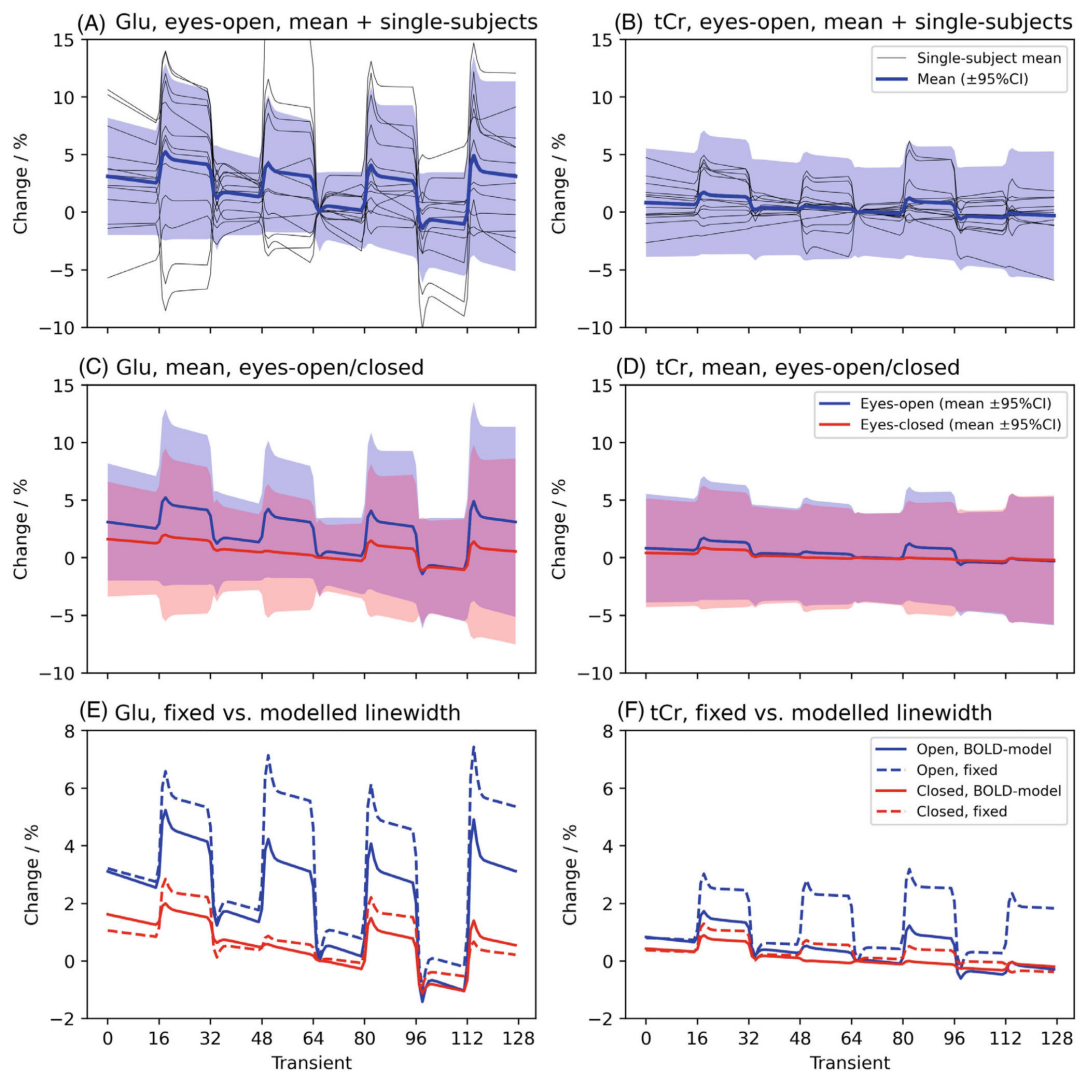
Results of the functional MRS (fMRS) in vivo validation for two metabolites glutamate, which is expected to increase with stimulation (Glu, left) and total creatine which is not expected to change (tCr, right). (A, B) Group-level mean and 95% CI (colored), and single-subject concentrations expressed as a percentage change relative to the middle time point. (C, D) Comparison of the group level means and 95% CIs for the stimulation (eyes-open) and control (eyes-closed) case. (E, F) Comparison of the results when using a model that incorporates the effect of BOLD on metabolite linewidths, to one with linewidths that are fixed across all timepoints (see Supporting Information S1). When fixed, spuriously large changes are observed during stimulation, including for non-modulating metabolites.

The original study found significant increases in glutamate, rising approximately 2% over baseline, no other assessed metabolite was found to change during stimulation. In this reanalysis, when BOLD-induced line narrowing was modeled in the GLM the same result was found, with only glutamate showing a statistically significant (at *p* < 0.05) increase (Figure 7 and Supporting Table S1). Glutamate was found to increase 3.1 ± 5.8% on average across all subjects and blocks (*p* = 0.04). A significant decrease in linewidth during the stimulation blocks was found for the eyes-open case (−0.18 ± 0.5 Hz), but not for the eyes-closed case (see Figure S9). This shows that the line narrowing was successfully modeled without introducing autocorrelation across the signal.

When the BOLD induced narrowing was not modeled (fixed linewidth model), and nor was it accounted for in processing by line broadening (i.e. expected BOLD line narrowing was not accounted for), an increase in glutamate upon stimulation was also found with increased magnitude, 4.4 ± 5.9%, and significance, *p* = 0.01, but also significant increases in tNAA (0.4 ± 1.0%), tCho (1.9 ± 2.2%), and tCr (1.9 ± 1.1%) were detected (Figure 7F). Full statistics are reported in Table S2.

### 3.5 CS5. dMRS: multi-direction diffusion encoding

#### 3.5.1 Setting

Simulated multi-direction diffusion weighted MRS data were used to demonstrate dynamic fitting enabling new data acquisition approaches for dMRS. By implementing dynamic fitting using a parameterized functional model of direction-dependent diffusion properties, acquisitions that have higher dynamic encoding resolution may be possible, when without dynamic fitting repeated sampling of each encoding would be needed for sufficient SNR. This case study also demonstrates the requirement and implementation of adequate fitting initialization using existing neuroimaging tools.

**Figure 8:**
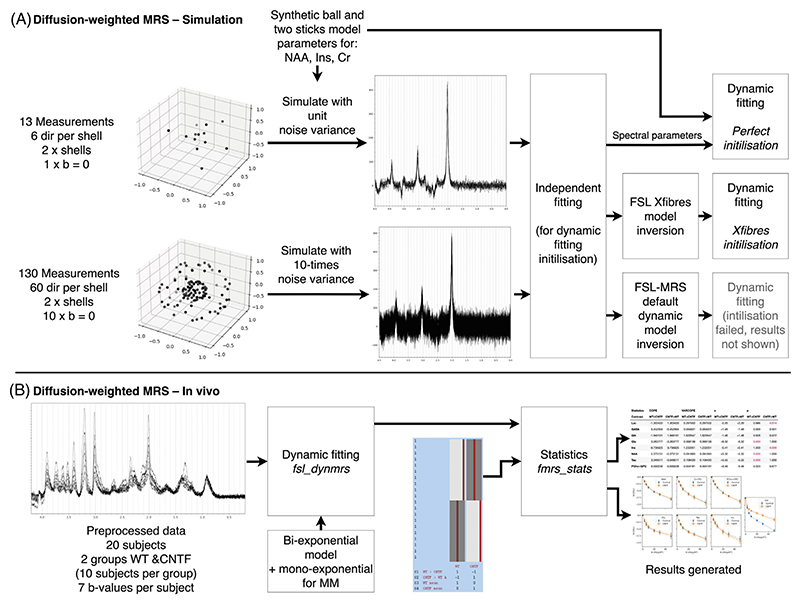
dMRS. (A) Schematic of the simulated analysis of multi-direction data using FSL-MRS’s dynamic approach. Time-matched data with different numbers of diffusion directions were analyzed, implementing a ball-and-two-sticks model into the spectral-dynamic fitting. Different fitting initialization approaches were trialed for each case. (B) Previously published multi-*b*-value dMRS data were reanalyzed using spectral-dynamic fitting, implementing a biexponential model (exponential for macromolecules). The group-level analysis using fmrs_stats was qualitatively compared to the published results. CNTF, cytokine ciliary neurotrophic factor.

Data were simulated (Figure 8A) for three metabolites: NAA, predominantly located neuronally; myo-inositol, predominantly located in glia; and creatine, a mix. The simulation was performed for a voxel with two crossing neuronal fiber populations, which is not present for glia. Thus, different simulated metabolites had different orientational diffusion dependence, i.e. different “fiber” orientation distribution function, fODF (simulations values in Supporting Information S1). NAA parameters were designed to mimic two crossing fiber populations, Ins as a predominantly spherical compartment (mimicking glia), and Cr was implemented as a mixture of the two. Synthetic data were simulated for a diffusion weighted sLASER sequence implementing two diffusion weighting approaches:

1 Six diffusion directions at two *b* values (*b* = 1 and 3 ms/μm^2^), plus *b* = 0, and,
2 Sixty diffusion directions at two *b* values (*b* = 1 and 3 ms/μm^2^), plus *b* = 0.

Simulated noise variance was set 10 times higher for condition #2, to simulate equal acquisition times (equivalently, the noise SD was set √10-times higher). Dataset #2 is what is commonly acquired for water diffusion for modeling crossing fibers (e.g. for tractography), whereas dataset #1 is closer to a dMRS design when repeated measurements are typically needed to increase SNR.

Dynamic fitting was implemented with a two-sticks and a ball diffusion model, which was also used to generate the synthetic data.^47^ The fitting was initialized with one of three approaches:

1 Inversion of the dynamic model using the independently fitted spectra (default in fsl_dynmrs),
2 With the ground truth parameters,
3 Using results from the independently fitted spectra passed to FSL’s xfibres routine.^47^ Xfibres is the core component of FSL’s Bayesian Estimation of Diffusion Parameters Obtained using Sampling Techniques for crossing fibers (BEDPOSTX), so is designed to estimate the equivalent problem for imaging data of water.

Quantitative assessment of fitting performance was made using Euclidian distance between “stick” vectors (scaled by “fiber” fraction) estimated to those generated from ground truth parameters. Vector direction was rectified before error calculation.

Code for this section is contained under ./dwmrs/1_simulation_dti (git.fmrib.ox.ac.uk/wclarke/fsl-mrs-dynamic-fitting/-/tree/master/dwmrs/1_simulation_dti).

#### 3.5.2 Outcome

**Figure 9:**
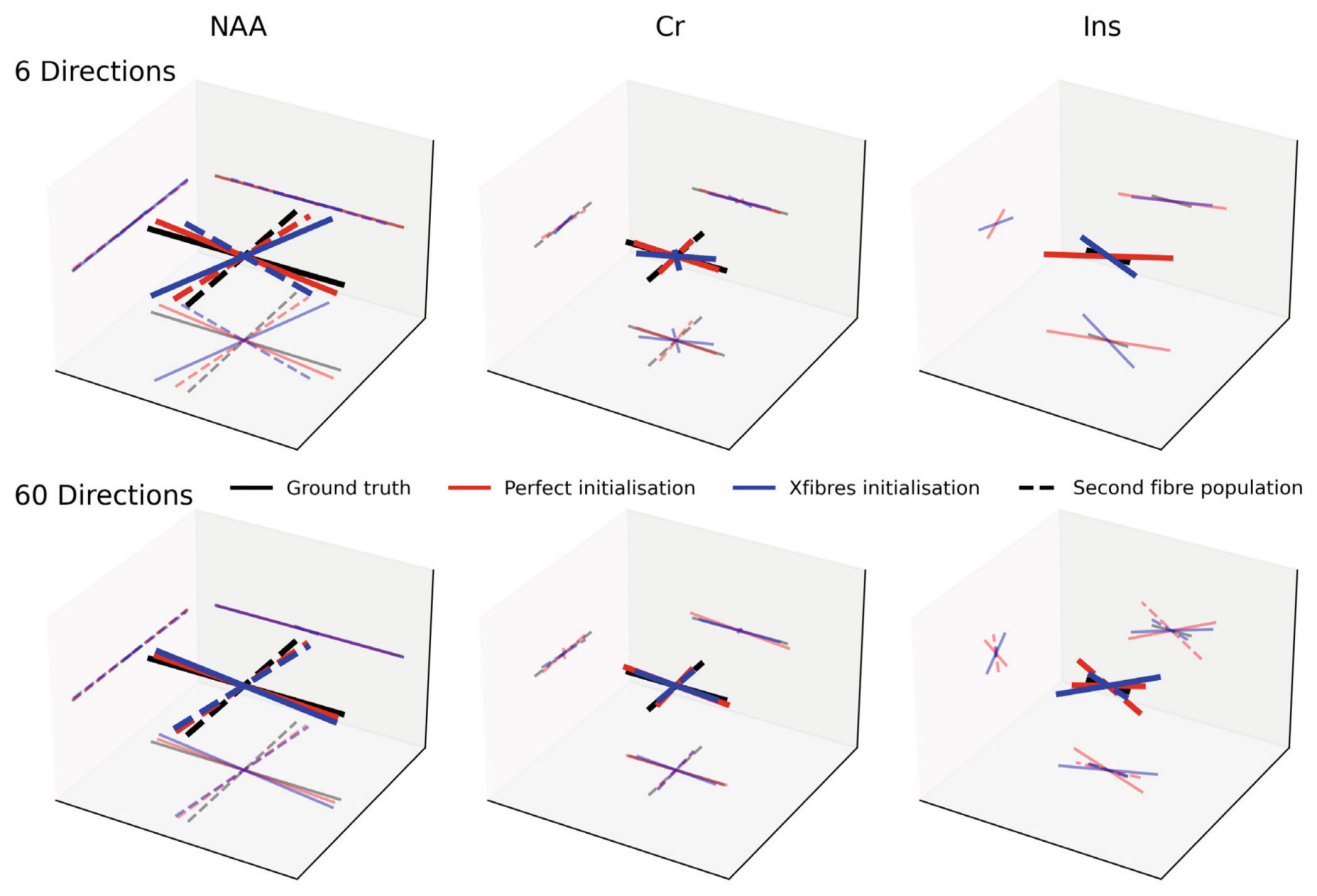
Fitting the ball and two-sticks model to simulated multi-direction diffusion data. This is a demonstration of the ability of the tool to simultaneously fit more complex diffusion models and spectral information. However, a good initialization point (provided by FSL’s xfibers tool) is required. Simulated data with more diffusion directions (but correspondingly lower spectral SNR) provide a better estimate of fiber directions than lower numbers of directions, which is required for stable spectral fits when no information is shared. The xfibres initialized fit, achievable on real data, is compared with an artificial perfect initialization approach (which requires the ground truth) and the ground truth. Each metabolite simulates a different cellular compartmentalization and therefore has a different ground truth.

Estimation of the simulated diffusion parameters was carried out for cases with 6 directions and 60 directions per diffusion shell, and using one of the three initialization strategies. For all metabolites, the 60 directions outperformed the 6 directions, and the perfect initialization outperformed the xfibers approach (Figure 9). The reduction in error between 6 directions to 60 directions (error measured as Euclidian distance between predicted and true fiber vectors) was 23% for the perfect initialization and 82% for xfibres. Fitting using the default initialization strategy (inversion of the model in FSL-MRS) failed in all cases due to a complex loss landscape resulting in multiple local minima, and the results are not shown.

### 3.6 CS6. dMRS: in vivo validation

#### 3.6.1 Setting

Dynamic fitting of diffusion-weighted MRS was demonstrated on a previously published dataset of a mouse model of “pure” astrocyte reactivity induced by injecting cytokine ciliary neurotrophic factor (CNTF).^48^ Two groups of 10 mice (control and CNTF) were scanned using a diffusion-weighted STE-LASER sequence,^49^ acquiring data at 7 *b*-values ranging from 0.02 to 50 ms/μm.^2^ Acquisition details are summarized in the Supporting Information S1.

Processed data were provided by the original study’s authors. Dynamic fitting implemented a bi-exponential model for metabolite concentrations, and a mono-exponential model for macromolecules. An exponential-plus-offset model was used for polynomial baseline terms, this accounts for decreasing baseline “roll” arising from unmodeled residual water signal, which decreases b-values increase. Without this baseline model either the baseline must be estimated independently for each *b*-value, increasing variability from a purely data-driven component of fitting, or a fixed value must be used, increasing bias. The spectral basis contained 20 metabolites, including empirically measured macromolecules (see Supporting Information S1).

Comparison was made to the original published results which implemented independent fitting using LCModel,^35^ and applied complex models of diffusion in randomly oriented cylinders.^6^

Code for this section is contained in under. /dwmrs/2_invivo (git.fmrib.ox.ac.uk/wclarke/fsl-mrs-dynamic-fitting/-/tree/master/dwmrs/2_invivo).

#### 3.6.2 Outcome

**Figure 10:**
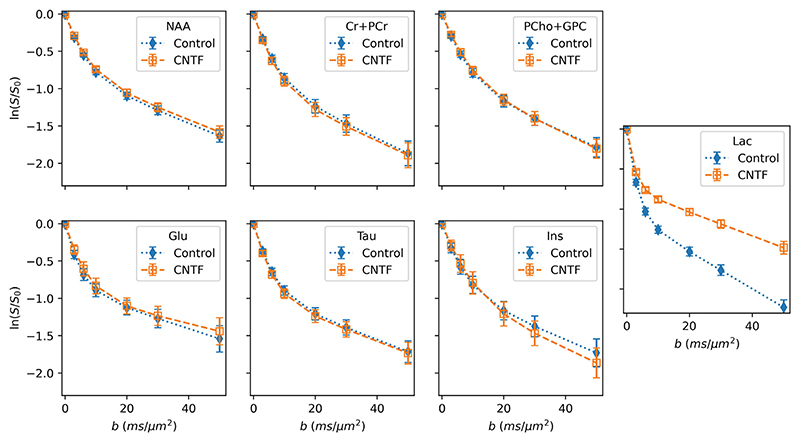
Dynamically-fitted concentrations of key metabolites in the in vivo dMRS validation. The results closely match the magnitude and direction of the original publication’s results, which found significant differences in myo-inositol (Ins) and lactate (Lac) diffusion properties between the control and CNTF cohorts. Changes in overall metabolite concentrations were also found (see Table S3), again matching the original publication.

The results of the proposed fitting approach accurately recapitulate the results of the original study, both looking at microscopic properties as measured using diffusion weighting (Figure 10) and overall metabolite concentrations (Supporting Information S1). Here, we find statistically significant differences in myo-inositol and lactate diffusion, and statistically significant differences in metabolite concentrations for Lac, Glu, Ins, NAA, Tau. The original publication found the same changes, apart from lactate concentration differences which did not reach statistical significance in the original, but we note that conservative Bonferroni correction was applied in the original.

## 4 Discussion And Conclusions

As demonstrated here, our universal dynamic MRS fitting tool allows simultaneous analysis of multiple, linked spectra. The tool implements a novel framework capable of handling several common types of dynamic MRS and incorporates methods for doing higher level statistics. We used the tool to replicate published results from fMRS and dMRS studies. We also numerically replicated theoretical results showing improvements in fitting uncertainty when using dynamic fitting approaches. This will help mitigate the low SNR inherent in dynamic spectroscopic approaches, improving target parameter precision. It will also enable greater dynamic resolution by permitting finer dynamic sampling despite the concurrent SNR reduction per shot.

In this work, we have demonstrated that, on in vivo data, the tool successfully replicates the results of published work from the original data, which used independent fitting approaches. On simulated data, where a ground truth is available, we demonstrated that the tool accurately estimates dynamic model parameters for diffusion weighted, functional, and edited spectroscopy experiments. Further, it was used to demonstrate an improvement in parameter estimation precision over independent fitting of edited spectroscopy. In addition, this work provides demonstration uses, and practical examples. Documentation has been developed beside the software tool and is available at fsl-mrs.com.

The tool also integrates into a larger neuroimaging toolbox, FSL, and uses standardized data formats, compatible with neuroimaging (NIfTI and NIfTI-MRS).^50,51^ By doing this, the tool will allow integration with existing neuroimaging methods (as demonstrated by the *fmrs_stats* tool) and use of well-validated MRI approaches, for example physiological noise regression in fMRS and mixed-effects group level modeling as shown in our dynamic fMRS results in this paper. To this point, in the toolbox, and in the examples shown, we have integrated existing models and software tools developed for analogous MRI techniques (fMRS/fMRI, dMRS/dMRI).

Dynamic fitting creates additional computational burden over independent fitting, needing longer computational times. The exact time is dependent on data size (number of transients and data points), number of fitting parameters (basis sets and dynamic fitting model), SNR, and computational hardware. However, in the cases presented here computation time for dynamic fitting ranges from 100% to 210% longer than independent fitting (the use in CS2 having the least increase, and CS4 the greatest), these numbers include the time required to initialise by performing an independent fit. The tools introduced include a command line interface to ease implementation on high-performance, distributed computer hardware.

Although our toolbox is designed to handle any type of dynamic spectroscopy, some practical limitations remain. Currently, the tool does not implement models which require dynamic parameters linked to more than one metabolite. For example, models where two or more metabolites interact dynamically. This limits applications to, for example, tracer experiments, such as used in hyperpolarized carbon-13 spectroscopy, where the dynamic modeling requires this level of flexibility. This limitation is however only a matter of implementation and will be addressed in future versions.

Finally, this work does not address an important caveat, which is identifying suitable dynamic models for linking across spectra. For example, in our fMRS analyses, we have approximated the response of metabolite concentrations to sensory stimulation using the canonical BOLD fMRI hemodynamic response function. While the BOLD response is well characterized in fMRI, it is not yet so in fMRS. Dynamic fitting using a suboptimal model may bias the results in ways that must be quantified in future studies. Similarly, there are many possible diffusion models already arising from dMRI,^52^ but these require adaptation for spectroscopy.^6^ It is likely that careful, long experiments with relatively low encoding space will be needed to establish a model using high SNR data, and often still using independent fitting. Custom acquisitions may be required, for example, repeated and jittered application of event related activation could be used to map out fMRS metabolic response functions.^53^ Although we note that dynamic fitting may allow exploration of more models, inappropriate for data acquired for high SNR with a small number of dynamic encodings.

Furthermore, there are myriad choices for the user to make during pre-processing, choice of fitting algorithm and model description: where the normal “static” spectral fitting parameters are extended by parameters describing the dynamic model. Changing many of these parameters can change the results, especially given the low SNR measurements we are fitting. This work does not start to address these choices, but the tool and framework presented provide a rigorous analysis and statistical platform to do model discovery and selection.

## Supplementary Material

S1

Supporting Information

## Data Availability

The code and data used in this work are available online. The underlying fitting code is available as part of FSL-MRS (version used 2.1.0, git hash a2dd6c38), this is available from fsl-mrs.com, https://git.fmrib.ox.ac.uk/fsl/fsl_mrs/, and a permanent record is available at https://doi.org/10.5281/zenodo.5910341. Some visualization tools shown in the Supporting Information S1 are only available in FSL-MRS version 2.1.16. The source code specifically for this work is available from https://git.fmrib.ox.ac.uk/wclarke/fsl-mrs-dynamic-fitting (specific git hash 11e4d66f), with a permanent record at https://doi.org/10.5281/zenodo.7956321. The original, unprocessed data for the in vivo fMRS and dMRS sections are available from https://doi.org/10.5281/zenodo.7950984. The specific fMRS fitting demonstration code is available from https://github.com/wtclarke/fsl_mrs_fmrs_demo (git hash a1e475c) with a permanent record at https://doi.org/10.5281/zenodo.7951648.
