# Supplementary material for: Universal dynamic fitting of magnetic resonance spectroscopy
Source: Magn Reson Med. Author manuscript; Available in PMC 2024 Oct 23. (PMC7616727; doi:10.1002/mrm.30001)
Supplement: Supporting Information [file EMS199450-supplement-Supporting_Information.pdf]

SUPPORTING INFORMATION

Additional supporting information may be found in the online version of the article at the publisher's website.

Supporting Information S1. Supporting information.

15222594, 2024, 6, Downloaded from https://onlinelibrary.wiley.com/doi/10.1002/mrm.30001, Wiley Online Library on [16/10/2024]. See the Terms and Conditions (https://onlinelibrary.wiley.com/terms-and-conditions) on Wiley Online Library for rules of use; OA articles are governed by the applicable Creative Commons License
